# The *Saccharomyces cerevisiae* transcriptome as a mirror of phytochemical variation in complex extracts of *Equisetum arvense* from America, China, Europe and India

**DOI:** 10.1186/1471-2164-14-445

**Published:** 2013-07-04

**Authors:** Rebekah Cook, James R Hennell, Samiuela Lee, Cheang S Khoo, Maria C Carles, Vincent J Higgins, Suresh Govindaraghavan, Nikolaus J Sucher

**Affiliations:** 1 Centre for Complementary Medicine Research, University of Western Sydney, Locked Bag 1797, Penrith, NSW 2751, Australia; 2 Ramaciotti Centre for Gene Function Analysis, School of Biotechnology and Biomolecular Sciences, University of New South Wales, Sydney, NSW 2052, Australia; 3 Network Nutrition Pty Limited, Level 1, 1 Richardson Place, North Ryde, NSW 2153, Australia; 4 Present address: Natural Sciences, Northern Essex Community College, 110 Elliot Street, Building E, Room 367, Haverhill, MA 01830, USA; 5 Present address: Science, Technology, Engineering & Math, Roxbury Community College, 1234 Columbus Ave, Roxbury Crossing, Boston, MA 02120, USA

**Keywords:** Herbal medicine, Transcriptomics, Phytochemistry, Chemometrics, Microarray, Functional genomics, Gene expression, Yeast, Phospholipid metabolism

## Abstract

**Background:**

Pattern-oriented chemical profiling is increasingly being used to characterize the phytochemical composition of herbal medicines for quality control purposes. Ideally, a fingerprint of the biological effects should complement the chemical fingerprint. For ethical and practical reasons it is not possible to test each herbal extract in laboratory animals or humans. What is needed is a test system consisting of an organism with relevant biology and complexity that can serve as a surrogate *in vitro* system. The purpose of this study was to test the hypothesis that the *Saccharomyces cerevisiae* transcriptome might be used as an indicator of phytochemical variation of closely-related yet distinctly different extracts prepared from a single species of a phytogeographically widely distributed medicinal plant. We combined phytochemical profiling using chromatographic methods (HPTLC, HPLC-PDA-MS/MS) and gene expression studies using Affymetrix Yeast 2.0 gene chip with principal component analysis and *k*-nearest neighbor clustering analysis to test this hypothesis using extracts prepared from the phytogeographically widely distributed medicinal plant *Equisetum arvense* as a test case.

**Results:**

We found that the *Equisetum arvense* extracts exhibited qualitative and quantitative differences in their phytochemical composition grouped along their phytogeographical origin. Exposure of yeast to the extracts led to changes in gene expression that reflected both the similarities and differences in the phytochemical composition of the extracts. The *Equisetum arvense* extracts elicited changes in the expression of genes involved in mRNA translation, drug transport, metabolism of energy reserves, phospholipid metabolism, and the cellular stress response.

**Conclusions:**

Our data show that functional genomics in *S. cerevisiae* may be developed as a sensitive bioassay for the scientific investigation of the interplay between phytochemical composition and transcriptional effects of complex mixtures of chemical compounds. *S. cerevisiae* transcriptomics may also be developed for testing of mixtures of conventional drugs (“polypills”) to discover novel antagonistic or synergistic effects of those drug combinations.

## Background

The notion that therapeutic effects of herbal medicines were due to the presence of a distillable “quintessence” popularized by Paracelsus and fellow alchemists some 450 years ago [1], morphed over time into the scientific hypothesis that pharmacological effects of herbal medicines are due to their content of plant-derived chemical compounds (mainly so called secondary metabolites) [2,3]. Research based on this hypothesis conducted over the last two centuries has led to the isolation and structural elucidation of some of the best-known drugs and has led to the creation of modern pharmacology and pharmacological therapy [2,4-7]. Herbal medicine, which can therefore rightfully be considered a progenitor of modern pharmacotherapy, has along the way been relegated to the sidelines and its continued popularity with the general public is viewed by many orthodox medical professionals at best as a useless but harmless anachronism that can be harnessed for its placebo effects or at worst as a harmful superstition with potentially lethal adverse effects that needs to be discouraged [8,9]. Herbal medicine will not regain a foothold in modern science-based medicine without clear evidence of therapeutic efficacy [10]. Such evidence has to come from testing in randomized, double blind clinical trials, which are considered as the “gold standard” of clinical medicine. In addition, successful demonstration of clinical effectiveness has to be complemented by an appropriate theoretical framework i.e. pre-clinical research providing a “mechanistic” basis for the observed clinical effects. The biological “target” of the drug and its function in the pathophysiology of the disease should be known [11-13]. A good drug is thought to act like Paul Ehrlich’s “magical bullet” that finds its target and in the process “destroys” the disease process [14].

While the immense success of modern pharmacotherapy is patently obvious, the recent shift to a preponderance of “chronic” rather “acute” diseases and the threat of empty drug “pipelines” has led to calls for a re-evaluation of the current practice of drug treatment and development. Combination therapy and so-called network and systems-based approaches to drug discovery are being advocated [4,15-18]. Instead of magic bullets for single targets, the future is thought to lie in the use of both single drugs or combinations of drugs with multi-target effects [19]. The wheel appears to have turned full circle. What has been regarded as its biggest problem, namely that herbal medicines contain a myriad of chemical components with potentially synergistic effects is now hailed as the basis of their purported therapeutic effectiveness in conditions, which have so far been refractory to single drug therapy [20,21]. Elucidation of the molecular effects and specificity of single ingredients in herbal extracts can be difficult, but the determination of the action of every single chemical component in phytochemically complex extracts has been essentially elusive.

Pattern-oriented chemical profiling (“fingerprinting”) is being increasingly used to gain a more comprehensive summary of herbal medicine quality [22-25]. In comparison, component-oriented single-marker based approaches (adapted from the mainstream pharmaceutical industry) do not account for the complex assortment of metabolites generally present in herbal medicine [22,23]. The pattern-oriented approach considers all detectable constituents of a given herbal material to establish a characteristic chemical profile without necessarily characterizing all chemical constituents or their precise biological effects. Ideally, a biological fingerprint should complement the chemical fingerprint [26]. Preferably, fingerprints of their biological effects should be obtained in the very organism that will be treated with the herbal extracts. For ethical and practical reasons, however, it is not possible to test each herbal extract in laboratory animals or humans. What is needed then, is a test system consisting of an organism with relevant biology but less complexity that can serve as a surrogate system.

The species of yeast known as *Saccharomyces cerevisiae (S. cerevisiae)* is arguably the best-understood eukaryotic organism. It is inexpensive to maintain, easy to grow and it is classified as a “generally recognized as safe” microorganism (it is commonly referred to as baker’s yeast). *S. cerevisiae* was at the very beginning of the “omics” revolution because it was both the first eukaryotic organism for which the whole genome sequence was completed [27] and the first organism that was studied at the whole transcriptome level. The nature and time course of the transcriptional response of *S. cerevisiae* to a large number of environmental changes have been characterized both qualitatively and quantitatively [28]. In addition, scientists have generated a collection of molecular-barcoded *S. cerevisiae* strains in which every single gene has been selectively deleted [29,30]. Approximately 45% of the *S. cerevisiae* genes are homologous to mammalian genes and hundreds of genes that have been linked to diseases in humans have orthologs in yeast [31].

In this study, we wanted to test the hypothesis that the *S. cerevisiae* transcriptome might be used as an indicator of phytochemical variation of closely-related yet distinctly different extracts prepared from a single species of a phytogeographically widely distributed medicinal plant. We chose the medicinal plant *Equisetum arvense* commonly known as “horsetail” as model herb and the single celled fungus *S. cerevisiae* as model organism for our experiments. *E. arvense* is distributed worldwide over the northern hemisphere [32]. *Equisetum* species and hybrids are well understood to possess extensive morphological, morphometric and chemotypical variation [33,34]*. E. arvense* is used in traditional medicine as diuretic, anti-inflammatory, antioxidant, antidiabetic, vasorelaxant and hemostatic [35-41]. It is also used in dozens of manufactured products claimed to promote general wellbeing and improve the health of hair, nails, skin, and bone. The main constituents found within *E. arvense* include alkaloids, flavonoids, phenylcarboxylic acids, sterols, styrylpyrones, and silica [33,42-44], which are thought to mediate the beneficial effects of this herbal medicine. Veit and co-workers distinguished two distinct chemotypes (chemodemes) of *Equisetum* based on their phenolic chemistry [33] but there is no information on variation of the biological and clinical effects due to these regional variants.

Here we report that the gene expression in *S. cerevisiae* exposed to globally sourced extracts of *E. arvense* reflected variation in their phytochemical composition*.* We have made the microarray data obtained in this study publicly available in the Gene Expression Omnibus (GEO) database of the National Center for Biotechnology Information of the USA (see Methods for details).

## Results

### Phytochemical fingerprinting

**Figure 1:**
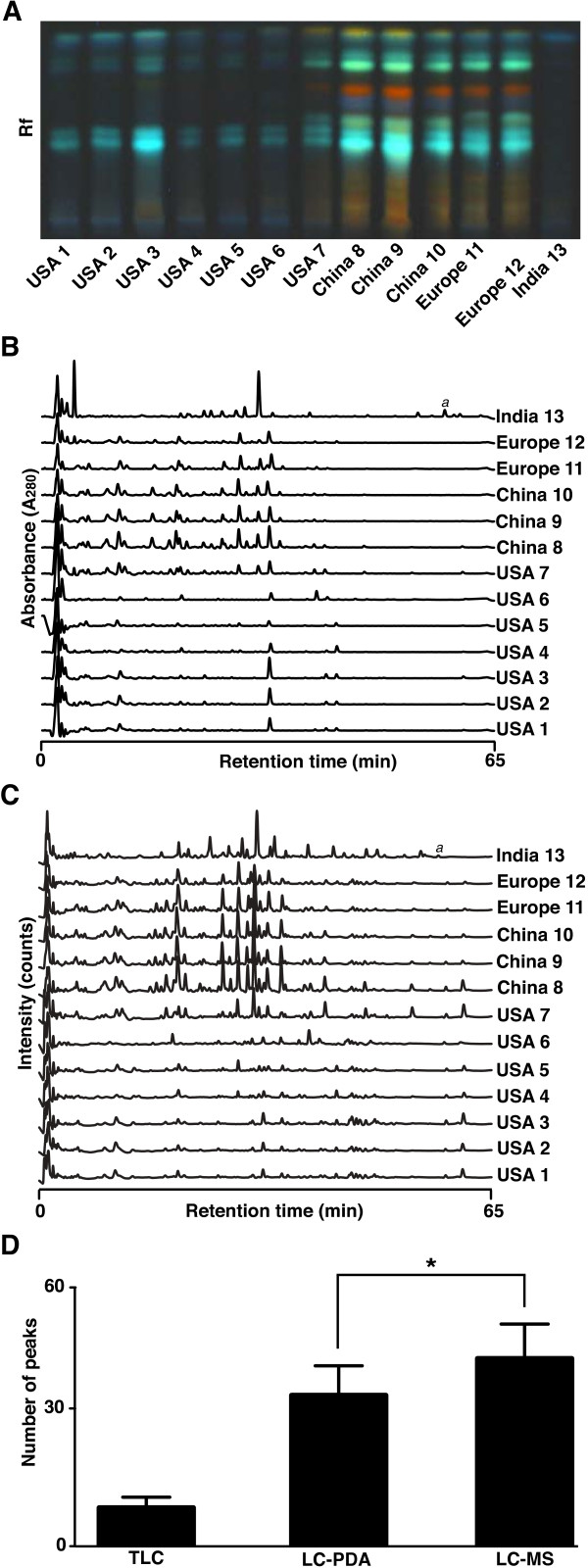
**Chromatographic characterization of the *E. arvense *extracts to identify quantitative and qualitative differences in phenyl carboxylic acid and flavonoid composition. (A)** A TLC plate developed with natural product / polyethylene glycol reagent, viewed under 366 nm UV light. **(B)** Stacked LC-PDA chromatograms observed at 280 nm. The letter “a” denotes dicaffeoyltartaric (chicoric) acid, which was only detected in the sample from India. Rf: relative front. **(C)** Stacked LC-ESI(−)-MS chromatograms. **(D)** The number of peaks detected in the TLC, LC-PDA and LC-MS chromatograms using the msProcess peak detection software. Means and standard deviations were calculated from biological replicates. * Represents a statistical significance of p < 0.05.

We used 3 standard chromatography-based separation and detection techniques of increasing complexity to characterize the phytochemical composition of aqueous extracts of *E. arvense* that were obtained from sources in the USA (*n* = 7; #1 - 7), China (*n* = 3; #8 - 10), Europe (*n* = 2; #11 - 12) and India (*n* = 1; # 13). The flavonoid and phenyl carboxylic acid high performance thin-layer chromatography (HPTLC) profile resolved on average 9 ± 3 peaks, but only a single peak was detected in the India sample (Figure 1A). The HPTLC profile clearly indicated a general quantitative difference in phenyl carboxylic acid and flavonoid concentration between the American and the European and Chinese samples.

Chromatograms generated using high performance liquid chromatography (HPLC) and detection using a photodiode array (PDA) set at 280 nm contained 35 ± 7 peaks, triple the number of constituents contained in the HPTLC profile (Figure 1B). The general trend in the variation of in phenyl carboxylic acid and flavonoid concentration along phytogeographical lines was similar to that obtained by HPTLC. The chromatograms furthermore exhibited clear qualitative differences between the samples, especially in regards to the Indian sample, which were detectable due to the increased sensitivity of the HPLC-PDA technique over HPTLC.

Next, we combined HPLC with mass spectrometry (MS) to analyze the samples. HPLC-MS detected on average 43 ± 8 peaks and revealed both qualitative and quantitative differences between the extracts as presented (Figure 1C).

Comparison of the different profiling techniques clearly illustrates that the discoverable complexity of the chemical composition of herbal extracts depends on the analytical technique used (Figure 1D).

The UV-Vis and mass spectra of peaks present in the LC-PDA and LC-MS chromatograms respectively were compared to the work by Veit et al [35] for tentative identification of some of the major chromatogram peaks (Table 1).

**Table 1:** **The tentative structural elucidation of several chemical constituents contained in the *E. arvense *samples**

**LC tR (min)**	**λ**_**max**_**by HPLC PDA (nm)**	**MS peaks (*m/z *)**	**Tentative ID**	**Reference**
5.6	241, 328	312 (100), 179, 149	Caffeoyl tartaric acid isomer	[45,46]
6.5	242, 327	312 (100)	Caffeoyl tartaric acid isomer	[45,46]
9.9	241, 327	312 (100), 225, 149	Caffeoyl tartaric acid isomer	[45,46]
11.4	241, 328	311 (100), 179, 148	Caffeoyl tartaric acid isomer	[45,46]
16.2	240, 323	367 (100), 225, 179, 135	Methyl caffeoylquinic acid	[45,46]
18.5	234, 315	336 (100), 295	Caffeoylshikimic acid isomer	[47]
19.8	237, 326	335 (100), 295, 179	Caffeoylshikimic acid isomer	[47]
24.6	231, 283	650 (100)	Quercetin or Protogenkwanin derivative	[48]
26.6	241, 342	448 (100), 319	Luteolin glucoside	[33,49]
28.7	265, 354	464 (100), 342, 300	Quercetin glucoside	[33,48]
29.3	255, 366	579 (100), 271	Apigenin 3-O-glucoside-7-O-rhamnoside	[33,50]
30.1	236, 261, 334	489, 463, 431 (100)	Apigenin glucoside	[33,49]
31.0	235, 269, 330	462, 410 (100)	Quercetin glucoside	[33,48]
31.4	235, 287	610, 301	Quercetin 3-O-glucoside-7-O-rhamnoside	[50]
32.9	238, 328	473, 311 (100), 178, 149	Dicaffeoyl tartaric acid	[51]
34.9	237, 261, 333	490, 445 (100)	Genkwanin glucoside isomer	[33,48]
41.1	288, 353	285 (100)	Kaempferol derivative	[33,48]
42.7	233, 270, 324	284 (100)	Genkwanin glucoside isomer	[33,48]
46.8	231, 288	302 (100)	Quercetin / Protogenkwanin	[48]

A representative example of how we elucidated the structure of dicaffeoyltartaric acid and a genkwanin acetylglucoside are presented in Additional file 1: Figure S1.

**Figure 2:**
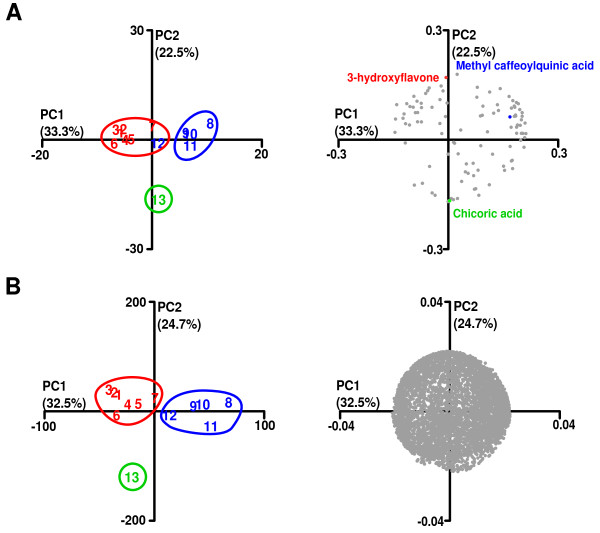
**Principal component analysis (PCA) of LC-ESI(−)-MS chromatographic peaks.** Scores plots are shown on the left, the corresponding loading plots on the right. The color and ellipses on the scores plots denote grouping obtained from *k*-NN with 3 specified clusters. The proportion of variance encompassed by each principal component is given in parentheses. **(A)** The scores plot (left panel) is based on the absolute amplitude of all 107 detected peaks, showing that the geographical origin of the extracts is primarily associated with the 3 specified groups (red = USA, extracts 1 to 7, blue = Europe, extracts 11 and 12 and China, extracts 8 to 10, green = India, extract 13). The loadings plot (right) highlights peaks that are representative of the grouping observed in the scores plot (3-hydroxyflavone for USA, methyl caffeoylquinic acid for Europe and China, dicaffeoyltartaric (chicoric) acid for India. **(B)** The scores plot (left panel) is of the 5,671 peak intensity ratios obtained from the 107 detected peaks using the rational of Tilton and colleagues [28] showing the geographical origin of the extracts is primarily associated with the 3 specified groups (red = USA, blue = Europe and China, green = India). The loadings plot (right) contained no significant information **(C)**.

Inspection of the HPTLC and HPLC chromatograms shown in Figure 1 appeared to suggest that the fingerprints obtained from the *Equisetum* extracts grouped largely according to their phytogeographical origin. The samples from Europe and China were more closely similar to each other then to the fingerprints of the Indian and American samples. American samples, in turn, appeared to be more closely related to each other then to the European and Chinese samples. In order to see whether the existence of subgroups within the data could be verified statistically, we used the multivariate statistical techniques of principal component analysis (PCA) and k-nearest neighbor (*k*-NN) clustering analysis to quantitatively characterize differences and similarities between the HPLC-MS fingerprints of the *E. arvense* extracts (Figure 2).

PCA essentially replaces the natural, albeit potentially subjective pattern recognition ability of the human brain by reducing the highly complex chromatogram data into a reduced data set, where each chromatogram is represented by a single point, which is then plotted in the so-called scores plot in relation to the first 2 principal components of the entire data set. We used *k*-NN to colorize the PCA, by highlighting samples that were classified into the 3 groups. PCA not only greatly reduces the complexity of the data it can also be used to determine which peaks and therefore phytochemicals underlie the observed differentiation into groups.

Figure 2A illustrates how the PCA (left panel) combined with *k*-NN clustering analysis (colored circles) grouped the chromatograms of the extracts along the lines of their phytochemical origin (USA, red; China / Europe, blue; India, green) with the sole exception of the European extract #12, which was grouped with the American extracts. Based on the similar proximity of chromatographic peaks in the loadings plot (right panel) to the sample groups in the scores plot, we were able to determine the peaks generally responsible for group differentiation. Three representative peaks have been highlighted in the same colors as the sample groups. For example, PCA identified dicaffeoyltartaric (chicoric) acid, which is highlighted in Figure 2A (right panel), as a differentiating factor for the Indian sample. The corresponding peak was indeed only detected in the Indian sample (Figure 1B and C, annotated by “a”).

Based on the work by Tilton et al [26] and their phytomics similarity index (PSI), we also conducted PCA based not on the intensities of the chromatographic peaks but on the ratio of each chromatographic peak intensity to each other within the same sample. That is *n* peak intensity values produce $\frac{n\left(n-1\right)}{2}\;$ unique ratio values. As illustrated in Figure 2B, PCA based on the intensity ratios combined with *k*-NN clustering analysis grouped extract #12 with the other European extracts and thus grouped all extracts according to their phytogeographical origin.

#### Radical scavenging capacity assays

Chemometric profiling of the *E. arvense* extracts demonstrated high variability in the flavonoid and phenyl carboxylic acid content. As flavonoids and phenolic acids have been reported to be effective free radical scavengers and antioxidants [52], we wondered to what degree the observed variation would be reflected in the radical scavenging capacity of the extracts [53]. The two main methods by which a compound can function as an antioxidant are hydrogen atom transfer (HAT) and electron transfer (ET) [53]. We therefore assessed the radical scavenging capacity of the *E. arvense* extracts using both HAT and ET mechanisms.

HAT reactions such as the oxygen radical absorbance capacity assay (ORAC) are kinetic based methods, whereby fluorescein and the antioxidant being measured compete for peroxyl radicals generated by the thermal decomposition of 2,2’-Azobis(2-amidinopropane) hydrochloride (AAPH) [54]. Therefore, competition by more potent antioxidant activity corresponds to slower fluorescein oxidation/degradation.

ET reactions such as those using 2,2-di(4-*tert*-octylphenyl)-1-picrylhydrazyl (DPPH) involve a redox reaction between the DPPH (oxidant) and the antioxidant compound being measured (reductant). DPPH is well suited for a rapid and simple antioxidant assay as it is commercially available and forms stable nitrogen radicals. In its oxidised form the DPPH has an intense purple color (λ_max_ 515 nm) and when it is reduced it becomes yellow (λ_max_ 320 nm), the color change being proportional to the antioxidant concentration.

Both the ORAC and DPPH methods use gallic acid as a reference for antioxidant capacity. That is, these assays measure how much better (or worse) the *E. arvense* extracts are at being antioxidants than gallic acid.

**Figure 3:**
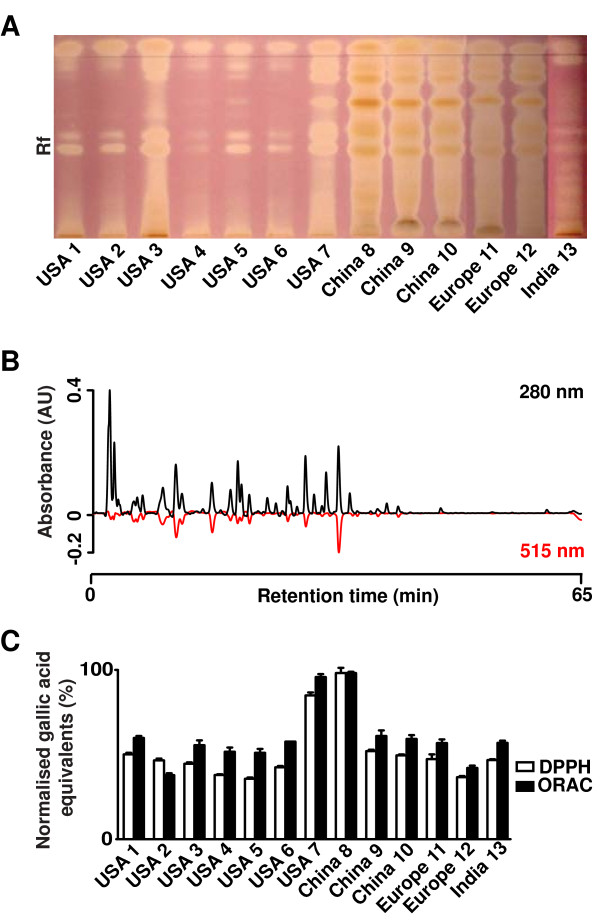
**Antioxidant activity of *E. arvense *extracts. (A)** A TLC plate developed in DPPH reagent, viewed under white light. Pink/purple regions are unreacted DPPH, lighter regions are where the DPPH radical has been scavenged by an antioxidant. After, dipping the TLC plate was left for 10 min for the reaction to proceed fully and intentionally “over-exposed” to reveal weak differences in radical scavenging. Rf: relative front. **(B)** A representative chromatogram of the China 8 sample using the on-line DPPH assay. The chromatogram at 280 nm (black line) is overlaid with the DPPH absorbance at 515 nm (red line). Compounds that have DPPH antioxidant activity are observed as a negative peak at 515 nm. **(C)** A comparison between the antioxidant abilities of each of the *E. arvense* extracts using the DPPH, FCR and ORAC assays.

As illustrated in Figure 3A, the Chinese and European extracts contained approximately 5 strongly antioxidant compounds. Peaks at 280 nm (as shown in Figure 1B; black line in Figure 3B) that have a DPPH radical scavenging capacity are identified by the corresponding decrease in DPPH absorbance measured at 515 nm (red line in Figure 3B). Overall, the ORAC and DPPH results were comparable, indicating that the flavonoids and phenyl carboxylic acids functioned in both the HAT and ET mechanisms. The China #8 and USA #7 samples showed the highest antioxidant capacity of the extracts (Figure 3C). This was unexpected and contrary to what was predicted by the phytochemical profiling, which indicated that the China and European extracts were similar to each other and distinct from the American extracts.

### Transcriptomic fingerprinting

**Figure 4:**
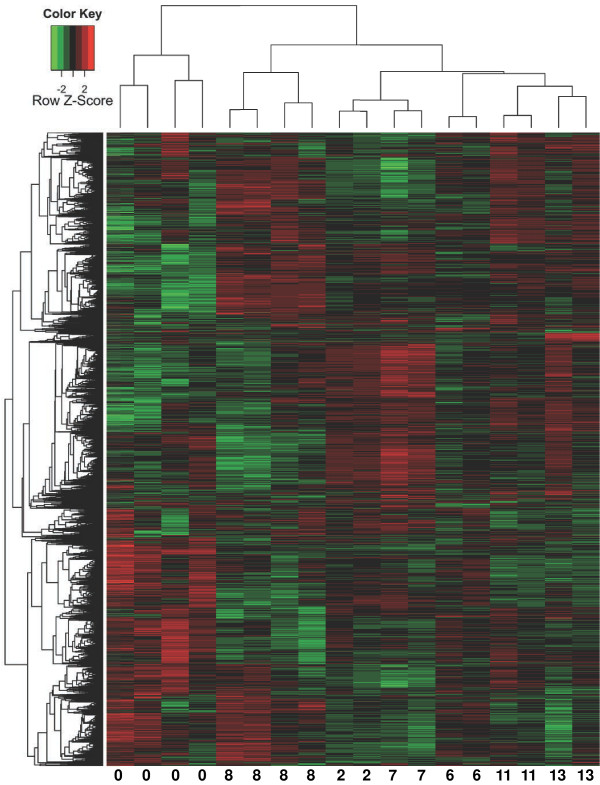
**Hierarchical clustering of gene expression values in four control and 14 extract treated samples.** Genes (rows) were clustered based on their distance in Pearson product–moment correlation (left dendrogram); arrays (columns) were clustered based on their distance in using Spearman rank correlation (top dendrogram). Numbers at the bottom of the figure indicate the identity of the arrays: 0, control, 8, China 8; 2, USA2, 7, USA7; 6, USA6; 11, Europe11; 13, India13. The expression levels (log2 transformed, see Methods for details) were scaled to the row mean. The color key indicates how the heat map colors are related to the standard score (z-score), i.e. the deviation from row mean in units of standard deviations above or below the mean.

The main goal of this study was to test the hypothesis that the *S. cerevisiae* transcriptome might be developed as an indicator of phytochemical variation of closely-related yet distinctly different extracts prepared from a single species of a phytogeographically widely distributed medicinal plant. We therefore exposed exponentially growing yeast cultures to representative extracts from each of the three groups identified by chemometric analysis (USA #2, *n* = 2 microarrays; USA #6, *n* = 2; USA #7, *n* = 2; China #8, *n* = 4, Europe #11, *n* = 2; India #13, *n* = 2) and the vehicle only (control, *n* = 4). We then harvested the yeast cells to extract total RNA for analysis using Affymetrix GeneChip® Yeast Genome 2.0 arrays. Figure 4 shows a raster plot (heatmap) of the averaged robust multi-array average (RMA)-corrected expression values of 5900 genes (rows) on 18 microarrays (columns). Genes and arrays were hierarchically clustered using distances calculated from their Pearson and Spearman correlation as indicated by the dendrograms on the left and top of the heatmap, respectively.

**Figure 5:**
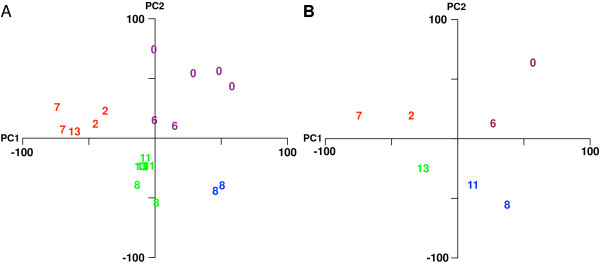
**PCA of the microarray results and comparison with the PCA of phytochemical data. (A)** Scores plot of the PCA of four control and 14 extract treated arrays. The numbers refer to the microarrays as follows: 8, China 8; 6, USA6; 11, Europe11, 13, India13; 2, USA2, 7, USA7. PC1 and PC2 account for 29 % and 24 %, respectively, of the total variance in the data. **(B)** Scores plot of the PCA of the mean expression values averaged over each set of arrays (control, *n* = 4, China #8, *n* = 4, Europe #11, n = 2, India #13, *n* = 2, USA #2, *n* = 2, USA #6, *n* = 2). PC1 and PC2 account for 36 % and 28 %, respectively, of the total variance in the data.

The clustering results indicate that the gene expression data not only distinguish the control samples from the extract treated samples, but also further differentiate between subgroups of the extract treated samples. We next performed PCA and *k*-NN clustering analysis of the gene expression data (Figure 5). Again, the analysis separated the samples into distinct clusters largely along phytogeographical origin and phytochemical variation, with the exception of USA sample #6, which was grouped with control samples (Figure 5A and B). Averaging of the expression values from each set of arrays (control, *n* = 4, China #8, *n* = 4, Europe #11, n = 2, India #13, *n* = 2, USA #2, *n* = 2, USA #6, *n* = 2) before PCA increased the signal to noise ratio of the data and therefore the diagnostic resolution (Figure 5B).

**Figure 6:**
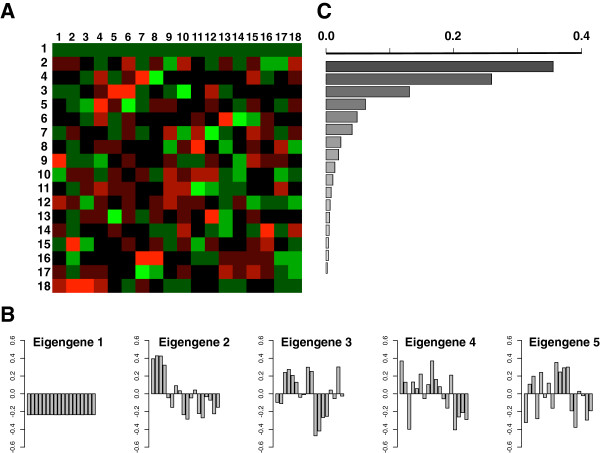
**SVD of the gene expression data. (A)** Heat map of 18 eigengenes of the entire data set. **(B)** Expression of the five most significant eigengenes in the 18 eigenarrays. Each bar corresponds to the expression level of the eigengene in each array (control: 1 to 4; USA 2: 5 and 6; USA 6: 7 and 8, USA 7: 9 and 10; China 8: 11 to 14; Europe 11: 15 and 16; India 13: 17 and 18 ) **(C)** Weights (eigenvalues) of the eigengenes and their relative contribution to the entropy of the data (d = 0.65) after filtering out eigengene 1, which did not differentiate between the arrays and can be considered to contribute only “noise” in the context of this study [56].

In the analysis of the gene expression data (as well as the phytochemical data), we used PCA and *k*-NN clustering as “diagnostic” tools with the goal to reduce the complexity of the data and to classify extracts into groups. PCA was performed by singular value decomposition (SVD) of the centered and scaled transpose of the data matrix [55]. SVD decomposes the data matrix (X) into three matrices commonly termed U, D and V. The columns of V (or rows of the transpose of V, V^T^) are referred to as the principal components of X [56,57]. Using terms more evocative for biologists, Alter and colleagues have referred to the rows of V^T^ as the eigengenes and the columns of U as eigenarrays [56]. The results of SVD of the data matrix without mean centering and scaling are illustrated in Figure 6. Inspection of the heatmap (Figure 6A) depicting the expression of the eigengenes (rows) in each array (columns) reveals that the expression of the first eigengene shows little variation between the arrays. This eigengene describes the contribution of gene expression that remains essentially constant. In contrast, the expression levels of the remaining eigengenes show clear differences both between the control and extract treated samples as well as differences between the extracts of different origin. Figure 6B illustrates the expression levels of eigengenes 1 to 5. Each bar represents the expression level of the respective eigengene in the arrays 1 to 18. It can be clearly seen that the second eigengene mainly represents the differences in gene expression between control (1 to 4) and treatment arrays (5 to 18). The eigengenes 3 to 5 highlight extract specific differences. The relative contribution of the eigengene 2 to 18 to the total variation in gene expression after eigengene 1 was filtered out is shown in Figure 6C.

**Figure 7:**
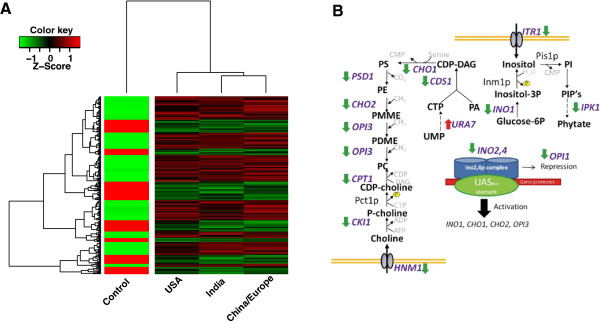
***S. cerevisiae *genes affected by treatment with *E. arvense *extracts. (A)** A heat map of 221 genes that were significantly different (p < 0.05 after Bonferroni correction) in a contrast between control (*n* = 4) and all other *E. arvense* arrays (*n* = 12). Samples were averaged to yield 3 groups according to the clustering in Figure 6A. Genes were clustered to highlight similarities between the different samples on the one hand and differences relative to control on the other hand*.* The expression levels are scaled to the row mean. The color key relates the heat map colors to the standard score (z-score), i.e. the deviation from row mean in units of standard deviations. **(B)** Schematic representation of pathway analysis of exemplary *S. cerevisiae* genes regulated by treatment with *E. arvense* extracts. Green arrows label downregulated genes and red arrows upregulated genes. The pathway enzymes differentially expressed were IPK1, inositol polyphosphate kinase; INO1, inositol-1 phosphate synthase; CDS1, phosphatidate cytidyltransferase; CHO1, phosphatidylserine synthase; PSD1, phosphatidylserine decarboxylases; CHO2, phosphatidylethanolamine N-methyltransferase; OPI3, Phospholipid N-methyltransferase, CPT1, cholinephosphotransferase; CKI1, choline kinase and URA7, CTP synthase. The important metabolites throughout this pathway are phosphatidate (PA), uridine monophosphate (UMP), cytidine triphosphate (CTP), cytidine diphosphate - diacylglycerol (CDP-DAG), inositol, inositol-3 phosphate (inositol-3P), glucose-6 phosphate (glucose-6P), phosphatidylinositol (PI), phosphatidyl inositol phosphates (PIP’s), phytate, phosphatidylserine (PS), phosphatidylethanolamine (PE), phosphatidylmonomethyl-ethanolamine (PMME), phosphatidyldimethylethanolamine (PDME), phosphatidylcholine (PC), cytidine diphosphate -choline (CDP-choline), phosphate choline (P-choline) and choline. The important cell membrane and cell wall (yellow bars) transporters in this pathway are the myo-inositol (ITR1) and choline/ethanolamine transporters (HNM1). Inset; an inositol and choline mediated regulation of INO2, INO4 and OPI1 genes; the products of which form a heterodimer that binds to an upstream activating site to regulate the transcription of the INO1, CHO1, CHO2 and OPI3 genes [61].

We further analyzed the microarray data using the default linear model included with the BioConductor “limma” package [58-60]. As robust linear modeling of microarray results generally requires 3 or more replicates per sample [59], we first contrasted all treatment arrays (USA, *n* = 4; Europe/China, *n* = 6; India, *n* =2) against the control arrays (*n* = 4) to generate a table of differential expression values ranked according to their Bonferroni-corrected *p*-values (*p* < 0.05). In order to simulate results using a fully automated process, the 2 arrays obtained upon exposure of yeast to sample USA #6 were not included in this analysis because USA #6 was grouped with the control samples in the PCA (Figure 5; inclusion of USA 6 did, however, not significantly change the results). Figure 7A shows a heatmap of 221 genes with significant changes in their expression levels compared to control in all 3 phytogeographical *E. arvense* groups that were identified by PCA. The *E. arvense* extracts elicited changes in the expression of genes involved in mRNA translation, drug transport, metabolism of energy reserves, phospholipid metabolism, and the cellular stress response.

Pathway analysis revealed that the pathways producing the major yeast phospholipids, phosphatidylserine (PS), phosphatidylethanolamine (PE), phosphatidylcholine (PC) and phosphatidylinositol (PI) were globally repressed upon exposure of yeast to *E. arvense* extracts independent of their phytochemical/phytogeographical grouping (Figure 7B). All of these phospholipids are synthesized through biological pathways after the transportation of choline and inositol into the yeast cell. The genes that encode the transporters of choline (*HNM1*) and inositol (*ITR1*) were both downregulated in the presence of *E. arvense* extracts. Once inside the cell, the proteins that convert choline into PC were downregulated (*CKI1* and *CPT1*). The genes that produce PS, PE and PC from cytidine triphosphate (CTP) and phosphatidic acid (PA) were downregulated (*CDS1, CHO1, PSD1, CHO2* and *OPI3*). In the inositol pathway, the gene responsible for the first step in the production of inositol from glucose-6 phosphate (*INO1*) was also downregulated. This global repression of the phospholipid synthetic genes was probably the result of the downregulation of the *INO2* and *INO4* genes (Figure 7B, inset). The proteins from these genes form a complex that has been shown to activate the expression of the *INO1*, *CHO1*, *CHO2* and *OPI3* genes [61]. Therefore, the absence of these proteins would result in decreased activation due to less binding to the conserved *cis*-acting UAS_*INO*_ element contained in their promoters. Repression of the *OPI1* gene is counter-intuitive to this theory since its role in the repression of the *INO2* and *INO4* genes would have thought it to be upregulated. However, White and colleagues [62] have shown that this action by the Opi1 protein is not brought about by the amount of protein but the activation of the protein itself.

The phospholipid precursors, inositol and choline have been shown to regulate the activity of a number of key enzymes within the yeast phospholipid biosynthetic pathway [63]. To investigate whether the *E. arvense* extracts contained inositol and choline, extracts from each area were analysed. A significant amount of inositol and choline was found to be present in all the *E. arvense* extract samples. For all samples the choline concentrations were similar, whereas, there was a large difference between the inositol concentrations (Table 2). Even though this difference was over 250-fold, the value of the lowest concentration of inositol was still higher than the concentration reported by Hirsch and Henry [64] that had an effect on yeast gene expression. These authors found that 75 μM (14 μg/mL) of inositol completely repressed the expression of the phospholipid synthesizing genes *INO1, CHO1, CHO2* and *OPI3.* The lowest concentration of inositol in our samples was 86 μM (Table 2). Thus, it is likely that the observed repression of genes in the phospholipid pathways in our experiments may have been due to the presence of high levels of inositol in the *E. arvense* extracts.

**Table 2:** Concentrations of inositol and choline in *E. arvense *samples* and concentration of inositol and choline^**+**^** added to yeast experimental treatments**

**Sample**	*Inositol mg/g	^**+**^**Inositol μg/mL**	*Choline mg/g	^**+**^**Choline μg/mL**
USA #2	219.83	549.57	13.22	33.05
USA #6	27.90	69.75	13.71	34.27
USA #7	10.27	25.67	7.69	19.22
CHINA #8	43.33	108.32	13.17	32.92
EUROPE #11	6.23	15.57	9.69	24.22
INDIA #13	75.70	189.25	5.17	12.92

**Figure 8:**
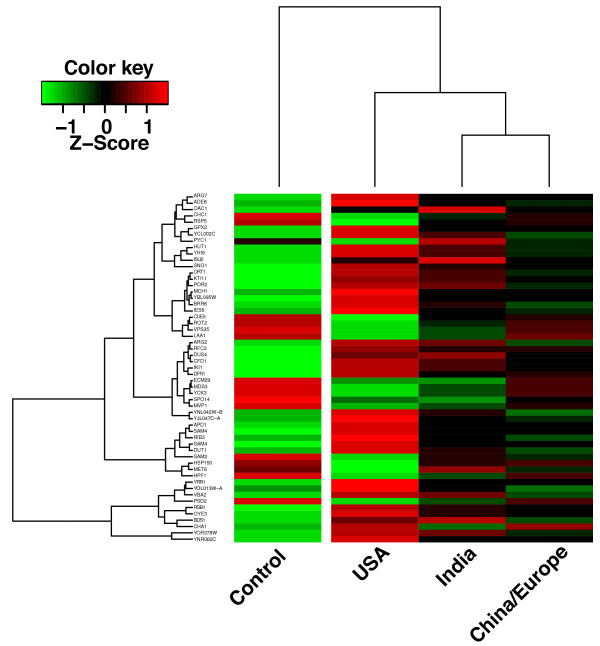
**An example of extract specific changes in gene expression.** A heat map of expression values of 55 significantly regulated genes (p < 0.05 after Bonferroni correction) in a contrast between control (*n* = 4) and the USA samples (*n* = 4) but not in the contrast between control (*n* = 4) and all other *Equisetum* samples (*n* = 12). Samples were averaged to yield 3 groups according to the clustering in Figure 6A. The expression values (log2 transformed) were scaled to the row mean. The color key relates the heat map colors to the standard score (z-score), i.e. the deviation from row mean in units of standard deviations above or below the mean. The gene symbols or (for transcripts without gene symbol) Ensembl identifiers [65] are listed.

To begin to identify extract specific changes in gene expression, we investigated which probes were selectively affected by the USA extracts. To this end, we performed an analysis using a linear model contrasting the control vs. USA and control vs. European/China samples. This analysis generated 2 data sets containing 150 probes in the Control-USA contrast, and 230 probes in the Control vs. Europe/China (*p* < 0.05 after Bonferroni correction). We then selected the probes that were contained only in the USA list (*n* = 55) and calculated the mean of their expression values in the control, China/Europe, India and USA sets of microarrays. Figure 8 shows the heatmap and hierarchal clustering of the results of this analysis. The map reveals the group specific differences between the samples. Preliminary pathway analysis of the data indicated that several of the identified genes are involved in amino acid metabolism and metabolism of nitrogen containing compounds but the group also contains several genes with as yet unidentified function. A detailed analysis along the lines illustrated by this example is in progress.

## Discussion

Using simple and hyphenated chromatography techniques we characterized extracts of *E. arvense* originating from America, China, Europe and India and found that they exhibited qualitative and quantitative differences in their phytochemical composition but similar antioxidant capacity. PCA combined with *k*-NN of the chromatographic data indicated that the phytochemical differences divided the extracts into three groups correlated with their phytogeographical origin from America, China/Europe or India. Supporting our hypothesis, analysis of whole genome microarray data with PCA and *k*-NN showed that the observed phytochemical grouping of the extracts was reflected in changes in gene expression in yeast exposed to the extracts, i.e. the *S. cerevisiae* transcriptome mirrored the phytochemical data. Importantly, PCA of the chromatographic and gene expression data is an unsupervised classification method that did not require the setting of more or less arbitrary thresholds (such “fold-increase or -decrease in gene expression) and does not discriminate between data points. In addition, the loadings plot can be used to pinpoint phytochemical peaks and genes that contributed to the differences between the groups. *k*-NN clustering analysis can be used to confirm quantitatively grouping of the extracts.

Statistical analysis of the gene expression data using a linear model revealed that the expression of 221 genes changed significantly upon exposure of *S. cerevisiae* to *E. arvense* extracts. Performing pathway analysis with these genes showed that the pathways producing the major *S. cerevisiae* phospholipids were globally repressed by all tested extracts independent of their phytochemical/phytogeographical grouping. This observation prompted us to quantify the inositol and choline content of the extracts, two essential components of the major yeast phospholipids. The data revealed that all extracts contained saturating amounts of these two essential nutrients.

Inositol and choline containing phospholipids play an important role in a large number of cellular processes in health and disease. Inositol is necessary for the synthesis of phosphoinositides, which function as lipid second messengers implicated in signal transduction and membrane trafficking [66]. Inositol has also been reported to be critical for the growth of keratinocytes [67] consistent with the use of *E. arvense* for the health of skin, hair and nails. Dietary administration of inositol has been claimed to have chemopreventive effects in rats [68]. Choline is not only required for the synthesis of phosphatidylcholine, lysophosphatidylcholine, choline plasmalogen, and sphingomyelin, which are essential components for all membranes, it is also a major dietary source of methyl groups (via the synthesis of S-adenosylmethionine) for methylation reactions that play major roles in lipid biosynthesis, the regulation of metabolic pathways, and detoxification [69]. While humans can produce inositol, choline is an essential nutrient. Yet the mean intake of choline for most people is far below the adequate intake [69]. The high choline content of the *E. arvense* extracts (more then twice the content in egg yolks, the most concentrated source of choline in the American diet [69]) is thus significant and supplementation of the diet with *E. arvense* might provide some general health benefits.

It is interesting to note that previous phytochemical studies of *Equisetum* mostly focused their general antioxidant properties and on phenolic compounds, sterols and the silica content of the herb [33,34,37,43,44,70-76]. To the best of our knowledge, the role of inositol and choline in relation to the beneficial effects of *E. arvense* has not been investigated previously. Our results immediately suggest further experiments. For example, it will be interesting to investigate whether exposure of yeast to saturating concentrations of choline and inositol alone will elicit similar changes in the expression of genes in the phospholipid synthesis pathway as observed in the present study. Exposure of yeast to the *E. arvense* extract fraction without inositol and choline will be an interesting complementary experiment.

Transcriptomic studies have previously been conducted both for the discovery of molecular effects of herbal medicines as well as quality control purposes [26,77-80]. In two previous studies, investigators combined phytochemical characterization of complex extracts from multiple herbs and microarray studies for what they called “bio-response fingerprinting” [26,80]. The purpose of the present study was diametrically opposite to that of the previous work. For example, Tilton and colleagues combined chemical fingerprinting, differential cellular gene expression and animal pharmacology studies followed by statistical pattern comparison to determine the similarity of the chemical and bio-response fingerprints among different manufactured batches of a multi herb preparation. These authors used the cellular assay as a “biological detector and the resulting genomic differential display profile after exposure to the botanical extract … (as) a sensitive and global biological metric …(to) validate batch *similarity* …” [26] (emphasis in italics is ours). Our aim, in contrast, was to test the hypothesis that the *S. cerevisiae* transcriptome might be used as an indicator of phytochemical variation of herbal extracts. Our data demonstrate that changes in the *S. cerevisiae* transcriptome reflected the phytochemical variation in complex extracts made from a single plant species. Thus, the yeast transcriptome can be used as a diagnostic tool for the classification of complex extracts even so the overwhelming majority of the genes did not show significant changes. While the diagnostic signals were relatively weak, they were picked out clearly by the PCA and cluster analyses. The functional significance of the observed changes for yeast remains to be established in future work.

## Conclusion

Together, the results of our study serve as a proof (or better demonstration) of principle and encourage further development of transcriptomic assays for the characterization of the biological effects of phytochemical variation of complex herbal extracts. Yeast transcriptomics may also be useful for testing of mixtures of conventional drugs (“polypills”) to discover novel antagonistic or synergistic effects of those drug combinations. Furthermore, it will be interesting whether or not observed changes in the transcriptome will be reflected at the proteome, interactome and metabolome [81-83]. Yeast is uniquely well positioned to serve as a model system for all types of “omics” studies.

We believe that the data presented here justify further exploration of this and similar (e.g. mammalian cell–based) systems of increasing yet manageable complexity useful for the development and testing of network and systems-based pharmacological therapies. In particular, the availability of yeast deletion and overexpression libraries offers the opportunity to study systematically the interaction between complex mixtures of small molecules and different genomes. The unparalleled progress in our understanding of the molecular basis of life especially in the second half of the 20^th^ century was driven by reductionism. There is an increasing number of scientists, however, who feel that complex systems may never be completely understood from the bottom up alone, especially in biological systems, and therefore advocate holism [84,85]. Obviously, single celled organisms such as *S. cerevisiae* cannot replace studies in multicellular organism but they can be used to discover molecular markers for monitoring in animal and human studies and are thus a first (“reductionist”) step towards holism in pharmacological studies of complex mixtures of chemical compounds.

## Methods

### Sources of E. arvense

LIPA Pharmaceuticals Ltd (NSW, Australia) provided us with authenticated dried *E. arvense* herb and non-standardized water extracts (USA, *n* = 7, 4:1 extract ratio, dicalcium phosphate excipient; China, *n* = 3, 5:1 extract ratio, glucose excipient; Europe, *n* = 2, 5:1 extract ratio, lactose monohydrate excipient; and India, *n* = 1, 4:1 extract ratio, dicalcium phosphate excipient). The authenticity of the extracts was established by phytochemical comparison against reference extracts prepared from authenticated *E. arvense* herbs with the traceability documents provided by each manufacturer and if dried raw herbs were available by genomic authentication.

#### Sample preparation

We removed the excipient from the commercial extracts in order to minimize sample variability due to the type of excipient used and the extract-to-excipient ratio. We weighed 4 g of each commercial extract into a 250 mL conical flask and added 250 mL of 80% aqueous methanol. We sonicated the solutions at 40 kHz for 1 h with occasional stirring and centrifuged the mixture at 4000 *g* for 5 min to pellet the insoluble excipient. We filtered the supernatant though a 0.45 μm PVDF syringe filter to remove any remaining particulates. To reduce the solution to dryness we rotary evaporated at 60°C to remove the methanol and then removed the remaining water by freeze drying for 12 h. We stored the product at 4°C when not in use.

#### Genetic authentication of the E. arvense samples

We extracted the genomic DNA from the dried aerial part of the plant and purified it using a Qiagen DNeasy mini plant mini kit (Victoria, Australia) according to the manufacturer’s instructions except we used water instead of buffer AE. The loci we chose for genomic authentication were the chloroplast genes maturase K (*matK*) and ribulose-1,5-bisphosphate carboxylase/oxygenase large subunit (*rbcL*) as specified by the Consortium for the Barcode of Life (CBoL) [86]. For the PCR amplification of *matK*, we used the primers ATACCCCATTTTATTCATCC in the forward direction and TACTTTTATGTTTACGAGC in the reverse direction as recommended by the Royal Botanic Gardens, Kew [87]. For the PCR amplification of *rbcL* we used the primers ATGTCACCACAAACAGAGACTAAAGC in the forward direction and GTAAAATCAAGTCCACCRCG in the reverse direction as recommended by CBoL [86]. We used the iProof high-fidelity DNA polymerase PCR kit from Bio-Rad Laboratories Inc. (NSW, Australia) for PCR amplification as per the manufacturer’s instructions for a 50 μL reaction with 35 cycles. The temperature program: initial denaturation 98°C, 60 s; denaturation 98°C, 30 s; annealing 53°C, 40 s; extension 72°C, 40 s; final extension 72°C, 5 min. PCR products we purified using the Qiagen QIAquick PCR Purification Kit according to the manufacturer’s instructions except that water is used instead of buffer AE. We sent our PCR products to The Australian Genome Research Facility Ltd. (NSW, Australia) for sequencing. We processed our data using the online program Geneious™ (Biomatters, Auckland, NZ).

We were successful in using both the matK and rbcL loci to authenticate the representative China, Europe and India E. arvense samples. We found the matK locus was better at differentiating E. arvense from the other Equisetum species than rbcL, with a BLAST search of GenBank® yielding between 97.3 - 99.9% (India and Europe respectively) identical sites to the E. arvense database entries using the matK products compared to 98.9 - 100% (Europe and India respectively) for rbcL. Although the percentage match using rbcL is higher, the percentages are equally shared with other Equisetum species, for example India shared the 100% match with both E. fluviatile and E. diffusum. Numerous single nucleotide polymorphisms (SNPs) are present in the matK sequence for the India sample, including an insertion between 465–472 bp not present in any other GenBank® entries. Nucleotide alignments of the China 8, Europe 11 and India 13 matK sequences against other species in the GenBank® database we have presented in Additional file 2: Figure S2. The sequences can be accessed through from GenBank® with the accession numbers JX392862-JX392864.

#### Phytochemical profiling

##### High performance thin layer chromatography (HPTLC)

We used a CAMAG (Muttenz, Switzerland) HPTLC system equipped with a sample applicator and visualization chamber with Merck (Darmstadt, Germany) silica gel 60 F_254_ HPTLC plates (20 cm × 10 cm). Our HPTLC profiling method was from Wagner et al. [88] using a mobile phase of ethyl acetate : formic acid : glacial acetic acid : water (100:11:11:26 mL).

We prepared working solutions of each extract by dissolving 50 mg of the purified sample in 1 mL 80% methanol. We then placed the solutions to sonicate briefly to dissolve the extract and filtered them using a 0.45 μm PVDF syringe filter. We applied 2 μl per lane to the plate.

To visualize the flavonoid and phenyl carboxylic acid profile, we developed the plate in natural products; diphenylboric acid 2-aminoethyl ester and polyethylene glycol 4000 (PEG) reagent and viewed at 366 nm.

To visualize the chemicals that scavenge the 2,2-diphenyl-1-picryl hydrazyl (DPPH) free radical, we developed the plate in DPPH reagent (200 μg/mL in ethanol) and visualized under white light. Chemicals that scavenge the DPPH radical appeared yellow.

#### HPLC–PDA and HPLC-ESI-MS/MS

We used a Varian (California, USA) LC system equipped with a Prostar 430 autosampler, ProStar 335 photodiode array detector (PDA) and 1200 L quadrupole MS/MS detector. We used an Alltech (Queensland, Australia) Prevail C18 column (150 mm × 4.6 mm, 5 μm) with a Phenomenex (California, USA) Security C18 guard column (2 mm × 4 mm, 5 μm).

We prepared working solutions of each extract by dissolving 50 mg of the purified sample in 1 mL 80% methanol. We sonicated the solution briefly to dissolve the extract and then filtered using a 0.45 μm PVDF syringe filter.

We generated LC-PDA and LC-MS profiles using a 10 μL injection volume and a mobile phase flow rate of 1 mL/min and a mobile phase consisting of 0.1% aqueous formic acid (mobile phase A) and acetonitrile (mobile phase B). The mobile phase profile was 10% B for 10 min and a linear increase to 50% B between 10–63 min. We washed with 100% B for 10 min and equilibrated with starting mobile phase for 10 min between each analysis.

We split the post-column flow to send 80% to the PDA and 20% to the mass spectrometer (MS) and acquired PDA chromatograms at 280 nm. The MS was acquired in negative electrospray ionization ((−)ESI) mode, scanning between 70*–*700 *m/z* using a nebulization gas (nitrogen) temperature of 400°C at 19 psi, needle voltage −3900 V at 15 μA, shield voltage −400 V, capillary voltage −100 V, and MS detector at −1700 V.

We analyzed the inositol and choline contents of the extracts using LC-MS in the (−)ESI mode with a selective ion monitoring (SIM) mode at 179 *m/z* and 103 *m/z* for inositol and choline, respectively. We set the nitrogen pressure to 20 psi at 250°C. The needle, capillary and detector voltage were −4500 V, -45 V and −1700 V respectively. For quantification, we used commercial standards. The limit of detection (LOD) being 3 μg/mL for each compound (three times method standard deviation (SD) and the limit of quantification (LOQ) was 10 μg/mL (ten times method SD).

We determined the flavonoid content using LC-PDA at 284 nm and used quercetin (3–300 μg/mL) as our standard to construct a calibration curve to quantify the flavonoid peaks. The total flavonoid content was 5 to 10% (w/w).

#### HPLC–DPPH-PDA

We visualized the chromatographic peaks that scavenge the DPPH radial by introducing DPPH reagent (40 μg/mL in 60% A and 40% B) into the post-column eluent using a third pump (0.6 mL/min) and reacting the solution in a coil (5.0 m × 0.5 mm) based on the work by Bandoniene et al. [89]. The PDA detector acquired at both 280 nm to monitor the chromatogram and 515 nm to monitor the degradation of the DPPH radical.

#### Antioxidant assays

We used a method adapted from Blois et al. and Molyneux et al. [90,91] to estimate the DPPH radical scavenging capacity of the *E. arvense* extracts compared to a gallic acid standard. We prepared all reagents in 80% aqueous methanol and the gallic acid standard curve by diluting a gallic acid stock (3 mM) to form 0.3, 0.6, 0.9 and 1.5 mM working standards. Then we prepared the samples by dissolving 1 mg of the extract in 10 mL of 80% aqueous methanol. For the reagent blank we used 80% aqueous methanol. In triplicate, we pipetted 180 μL of the DPPH reagent (250 μM) into each microtitre plate well and then 20 μL of either working standard, sample or blank to make a total volume of 200 μL. To correct for sample absorbance (i.e. absorbance not due to DPPH), we prepared sample blanks in triplicate by adding 180 μL of 80% aqueous methanol to the well and 20 μL of sample. We vortexed the plate at 700 rpm for 30 min in the dark prior to measuring absorbance at 515 nm. The sample antioxidant scavenging capacity is reported as the gallic acid equivalent.

#### Oxygen radical absorbance capacity assay

We performed the oxygen radical absorbance capacity (ORAC) assay in order to measure the ability of the *E. arvense* extracts to protect fluorescein from degradation by peroxyl radicals using the method described in the BMG LABTECH application note 148 [92] using Trolox® as the reference standard. We prepared all reagents in pH 7.4 phosphate buffer (10 mM). To construct the Trolox® standard curve we diluted the Trolox® stock (200 μM) to 12.5, 25, 50 and 100 μM working standards. We prepared samples by dissolving 1 mg of extract in 10 mL of 80% aqueous methanol. We used aqueous methanol (80%) as the reagent blank. For analysis, we used 150 μL fluorescein (10 nM) and 25 μL of either Trolox® standard, sample or blank in each microtitre plate well which was then vortexed for 30 min at 37°C. Rapidly we added 25 μL of the radical generator 2,2’-azobis(2-amidinopropane)dihydrochloride (AAPH, 240 mM) to each well and measured the plate every 90 s (excitation 485 nm, emission 520 nm). We compared the area under the signal degradation curves of the samples to the Trolox® standard and the results were given as Trolox® equivalents.

#### Yeast transcriptomics

We used the BY4743 (*Saccharomyces cerevisiae*) yeast strain (*MATα/MATα his3*∆*1/his3*∆*1 leu2*∆*0/leu2*∆*0 met15*∆*0/MET15 LYS2/lys2*∆*0 ura3*∆*0/ura3*∆*0*) [93,94] for our experiments. We grew the yeast to log phase overnight at 30°C in minimal medium prepared the same as Bell et al. [95] except that 20 mg/mL uracil was added. We treated 25 mL of the log phase replicate cultures (OD_600_ between 0.5-1.0) with dried *E. arvense* extracts at a concentration of 2.5 mg/mL in the media for 20 min. We conducted preliminary experiments to determine the optimal dose of *E. arvense* extract required for a significant effect on yeast gene expression. We tested dosages of 0.01, 1.0, 2.5, 5.0 and 10 mg/mL in the media using China 8 extract as a representative sample. We also obtained a concurrent growth curve with each microarray experiment. We covered a range of CHINA-8 concentrations from 0 mg/mL to 10 mg/mL and there was no affect on yeast growth at any of the concentrations. We chose a concentration of 2.5 mg/mL for the final study since 0.01 and 1.0 mg/mL produced little change in the gene expression profile of the yeast, whereas 2.5 mg/mL resulted in approximately 1.5% of the genes in the genome being differentially expressed by more than 2-fold. The extracts analyzed and numbers of biological replicates performed were: USA 2 (*n* = 2), USA 6 (*n* = 2), USA 7 (*n* = 2), China 8 (*n* = 4), Europe 11 (*n* = 2), India 13 (*n* = 2) and non-treated control (*n* = 4). We then harvested the treated yeast cells by centrifugation at 4000 *g* for 5 min. Cell pellets were snap frozen in liquid nitrogen and stored at −80°C prior to RNA isolation.

### Isolation of yeast RNA, reverse transcription, labeling and hybridization for microarray analysis

We used a method adapted from Winzeler et al. [29] to extract total RNA from *S. cerevisiae*. We mechanically disrupted the frozen cell pellets and extracted total RNA using TRIzol™ (Invitrogen, Australia) reagent according to the manufacturer’s instructions. We purified the total RNA using RNeasy spin columns (Qiagen, Australia); assessed RNA quality using an Agilent Bioanalyzer 2100 (California, USA) and quantified the RNA using a Thermo Scientific NanoDrop™ 1000 spectrophotometer (California, USA). We submitted our purified RNA samples to the University of New South Wales Ramaciotti Centre for Gene Function Analysis (NSW, Australia) for RNA transcription, labeling, hybridization, washing and scanning of the microarray slides. We used Affymetrix (California, USA) GeneChip® Yeast Genome 2.0 Arrays (containing 25-mer probes with 11 probe pairs per sequence for 5841 *Saccharomyces cerevisiae* transcripts and 5031 *Schizosaccharomyces pombe* transcripts). The microarray results (*E. arvense*-treated *n* = 14, control *n* = 4) can be accessed at Gene Expression Omnibus (GEO) http://www.ncbi.nlm.nih.gov/geo/query/acc.cgi?acc=gse24888.

#### Statistical analysis

We used the ‘R Project for Statistical Computing’ [96] for most of our data processing and statistical analysis. Specific packages used with R are detailed below. The R code for both the chemometric and biometric analyses are available upon request from the corresponding author.

#### Chemometric analysis

We used the package ‘msProcess’ [97] to ‘correct’ chromatograms by removing instrumental noise, baseline drift, identifying peaks, removing peak retention time variations between samples and to quantify peak height.

We used principle component analysis (PCA) together with *k*-nearest neighbor (*k*-NN) clustering analysis to cluster samples and highlight the chemicals potentially responsible for these differences using the ‘stats’ package included with R. Firstly, we conducted PCA on the corrected chromatograms and the results plotted using the first 2 principal components (PCs). We then applied *k*-NN to the first 2 PCs in order to identify samples that cluster together. Three groups were specified for the *k*-NN based on the country of origin of the sample: 1) USA, 2) China / Europe and 3) India. We compiled the group-specific peaks and their corresponding UV and MS spectra and compared them with those in the literature [33] to tentatively identify the compounds.

Using the chromatogram correction technique outlined above, we also determined the average number of peaks detected using standard techniques commonly used in the herbal extract industry including HPTLC, HPLC-PDA and HPLC-MS to estimate their information content. To determine the statistical significance (*p* < 0.05) between the analytical techniques, we used one-way ANOVA with a Tukey post-test using GraphPad Prism 5.0d for Mac OS X [98].

### Biometric analysis

We used theBioconductor [60,99] packages ‘affy’ [100], ‘affyPLM’ [101], ‘altcdfenvs’ [102], ‘annaffy’ [103], ‘limma’ [58], ‘yeast2cdf’ [104], and ‘yeast2.db’ [105] for yeast microarray analysis (reading the microarray *.cel files, assessing the files for RNA degradation, relative log expression, normalized unscaled standard error and spatial artifacts). We processed the probe expression values using the robust multi-array average (RMA) model for convolution background correction, quantile normalization and summarization [106,107]. We performed PCA on the averaged RMA-corrected expression values using the function prcomp in the R ‘stats’ package and SVD using the function svd in the R ‘base’ package.

### Pathway analysis

Statistical analysis of our microarray data resulted in a list of differential genes that were common between all *E. arvense* samples. We used 3 complementary web-based platforms to evaluate our gene sets and ascertain the cellular and molecular pathways affected in the yeast response to treatment. Principally, we used Funspec [108] (*p*-value cut-off 0.01) to analyse our gene list. Funspec compiles information to output a classification summary of genes and gene families that are enriched in the ontology of 1) cellular components, 2) molecular functions and 3) biological processes. Secondly, we conducted pathway mapping of differentially expressed genes to the annotation terms within the Kyoto Encyclopaedia of Genes and Genomes (KEGG) [109]. This process identified pathways and the functional locations of genes within pathways. Thirdly, we used the Saccharomyces Genome Database (SGD) [110] to obtain gene specific information linking additional genes from our data set to the pathway analysis.

## Abbreviations

AAPH: 2,2′-Azobis(2-amidinopropane) hydrochloride; CBoL: Consortium for the Barcode of Life; CDP-choline: Cytidine diphosphate-choline; CDP-DAG: Cytidine diphosphate – diacylglycerol; CDS1: Phosphatidate cytidyltransferase; CHO1: Phosphatidylserine synthase gene; CHO2: Phosphatidylethanolamine N-methyltransferase; CKI1: Choline kinase gene; CPT1: Cholinephosphotransferase gene; CTP: Cytidine triphosphate; DPPH: 2,2-di(4-*tert*-octylphenyl)-1-picrylhydrazyl; glucose-6P: Glucose-6 phosphate; HNM1: Choline/ethanolamine transporter gene; HPLC–DPPH-PDA: High performance liquid chromatography with introduction of DPPH into the post-column eluent using a third pump coupled with photo array detector; HPLC–PDA: High performance liquid chromatography coupled with photo array detector; HPLC-ESI-MS/MS: High performance liquid chromatography coupled with electrospray ionization tandem mass spectrometry; HPTLC: High performance thin layer chromatography; INO1: Inositol-1 phosphate synthase gene; inositol-3P: Inositol-3 phosphate; IPK1: Inositol polyphosphate kinase gne; ITR1: Myo-inositol transporter gne; KEGG: Kyoto Encyclopaedia of Genes and Genomes; k-NN: *k* nearest neighbor clustering analysis; LC tR: Liquid chromatography retention time; matK: Maturase K; OPI3: Phospholipid N-methyltransferase; ORAC: Oxygen radical absorbance capacity assay; PA: phosphatidate; PC: Phosphatidylcholine; PCA: Principal component analysis; P-choline: Phosphate choline; PDME: Phosphatidyldimethylethanolamine; PE: Phosphatidylethanolamine; PI: Phosphatidylinositol; PI: Phosphatidylinositol; PIP: Phosphatidyl inositol phosphate; PMME: Phosphatidylmonomethyl-ethanolamine; PS: Phosphatidylserine; PSD1: Phosphatidylserine decarboxylases gene; rcbL: Ribulose-1,5-bisphosphate carboxylase/oxygenase large subunit gene; PSI: Phytomics similarity index; RMA: Robust multi-array average; SIM: Single ion monitoring; UMP: Uridine monophosphate.

## Competing interests

The authors declare that they have no competing interests.

## Authors’ contributions

RC, JRH, and SL are joint first authors listed in alphabetical order. RC conducted the transcriptomic experiments and pathway analysis and analyzed the transcriptomic data together with VJH; JRH, SL, CK, SG conducted and analyzed the phytochemical experiments; JRH performed the multivariate statistical analysis using R; MCC performed the genomic authentication experiments; SG and NJS designed the study; NJS wrote the manuscript with participation of the co-authors. All authors read and approved the final manuscript.

## Supplementary Material

Additional file 1: Figure S1 Tentative structural elucidation of dicaffeoyltartaric (chicoric) acid and a genkwanin acetylglucoside using LC-ESI(-)-MS and LC-PDA. (A, B, C) the ESI(-)-MS, UV spectrum and proposed fragmentation pattern respectively of the dicaffeoyltartaric acid peak. (D, E, F) the ESI(-)-MS, UV spectrum and proposed fragmentation pattern respectively of the Genkwanin acetylglucoside peak, possibly 4 or 5 -O-(6-acetyl glucoside). Click here for file

Additional file 2: Figure S2 DNA bar codes of the original plant material used to produce the China 8, Europe 11 and India 13 extracts compared to other *Equisetum* species entries in the GenBank database. Differences between the sequences are marked with a colored box. Click here for file

## References

[B1] Ball P The devil's doctor : Paracelsus and the world of Renaissance magic and science, 1st American edn 2006 New York: Farrar, Straus and Giroux

[B2] Hamburger M Hostettmann K: 7 Bioactivity in plants: the link between phytochemistry and medicine. Phytochemistry 1991 30 12 3864 3874

[B3] Kinghorn AD Biologically active compounds from plants with reputed medicinal and sweetening properties J Natural Products 1987 50 6 1009 1024 10.1021/np50054a002 3327919

[B4] Lowe JA Jones P Wilson DM Network biology as a new approach to drug discovery Current opinion in drug discovery & development 2010 13 5 524 526 20812143

[B5] Newman DJ Cragg GM Snader KM The influence of natural products upon drug discovery Nat Prod Rep 2000 17 3 215 234 10.1039/a902202c 10888010

[B6] Newman DJ Cragg GM Snader KM Natural products as sources of new drugs over the period 1981–2002 J Natural Products 2003 66 7 1022 1037 10.1021/np030096l 12880330

[B7] Kinghorn AD The discovery of drugs from higher plants Biotechnology 1994 26 81 108 7749315 10.1016/b978-0-7506-9003-4.50010-1

[B8] Ernst E Herbal medicines–they are popular, but are they also safe? European J Clin Pharmacology 2006 62 1 1 2 10.1007/s00228-005-0070-2 16341855

[B9] Ernst E Herbal medicines: balancing benefits and risks Novartis Found Symp 2007 282 154 167 discussion 167–172, 212–158 17913230 10.1002/9780470319444.ch11

[B10] Ernst E Herbal medicines: where is the evidence? BMJ 2000 321 7258 395 396 10.1136/bmj.321.7258.395 10938031 PMC1127780

[B11] Kinghorn AD Chai HB Sung CK Keller WJ The classical drug discovery approach to defining bioactive constituents of botanicals Fitoterapia 2011 82 1 71 79 10.1016/j.fitote.2010.08.015 20804827

[B12] Heath G Colburn WA An evolution of drug development and clinical pharmacology during the 20th century J Clin Pharmacol 2000 40 9 918 929 10.1177/00912700022009657 10975064

[B13] Flower A Witt C Liu JP Ulrich-Merzenich G Yu H Lewith G Guidelines for randomised controlled trials investigating Chinese herbal medicine J Ethnopharmacology 2012 140 3 550 554 10.1016/j.jep.2011.12.017 22210103

[B14] Ehrlich P On immunity with special reference to the relationship between distribution and action of antigens 1908 Therapy: Experimental Researches on Specific 107

[B15] Arrell DK Terzic A Network systems biology for drug discovery Clin Pharmacol Therapeutics 2010 88 1 120 125 10.1038/clpt.2010.91 20520604

[B16] Pujol A Mosca R Farres J Aloy P Unveiling the role of network and systems biology in drug discovery Trends Pharmacol Sci 2010 31 3 115 123 10.1016/j.tips.2009.11.006 20117850

[B17] Hopkins AL Network pharmacology: the next paradigm in drug discovery Nat Chem Biol 2008 4 11 682 690 10.1038/nchembio.118 18936753

[B18] Azmi AS Wang Z Philip PA Mohammad RM Sarkar FH Proof of concept: network and systems biology approaches aid in the discovery of potent anticancer drug combinations Mole Cancer Therapeutics 2010 9 12 3137 3144 10.1158/1535-7163.MCT-10-0642 PMC3058926 21041384

[B19] Csermely P Agoston V Pongor S The efficiency of multi-target drugs: the network approach might help drug design Trends Pharmacol Sci 2005 26 4 178 182 10.1016/j.tips.2005.02.007 15808341

[B20] Ulrich-Merzenich G Panek D Zeitler H Wagner H Vetter H New perspectives for synergy research with the "omic"-technologies Phytomedicine: international journal of phytotherapy and phytopharmacology 2009 16 6–7 495 508 19428231 10.1016/j.phymed.2009.04.001

[B21] Wagner H Synergy research: a new approach to evaluating the efficacy of herbal mono-drug extracts and their combinations Nat Prod Commun 2009 4 2 303 304 19370944

[B22] Mok DKW Chau F-T Chemical information of Chinese medicines: A challenge to chemist Chemom Intell Lab Syst 2006 82 1–2 210 217

[B23] Zeng Z Chau FT Chan HY Cheung CY Lau TY Wei S Mok DK Chan CO Liang Y Recent advances in the compound-oriented and pattern-oriented approaches to the quality control of herbal medicines Chinese Med 2008 3 9 10.1186/1749-8546-3-9 PMC2531114 18680568

[B24] Kong WJ Zhao YL Xiao XH Jin C Li ZL Quantitative and chemical fingerprint analysis for quality control of rhizoma Coptidischinensis based on UPLC-PAD combined with chemometrics methods Phytomedicine: international journal of phytotherapy and phytopharmacology 2009 16 10 950 959 10.1016/j.phymed.2009.03.016 19553096

[B25] Liu EH Qi LW Li K Chu C Li P Recent advances in quality control of traditional Chinese medicines Combinatorial chemistry & high throughput screening 2010 13 10 869 884 10.2174/138620710793360301 20883191

[B26] Tilton R Paiva AA Guan JQ Marathe R Jiang Z van Eyndhoven W Bjoraker J Prusoff Z Wang H Liu SH A comprehensive platform for quality control of botanical drugs (PhytomicsQC): a case study of Huangqin Tang (HQT) and PHY906 Chinese Med 2010 5 30 10.1186/1749-8546-5-30 PMC2940884 20727161

[B27] Goffeau A Barrell BG Bussey H Davis RW Dujon B Feldmann H Galibert F Hoheisel JD Jacq C Johnston M Life with 6000 genes Science 1996 274 5287 546 567 10.1126/science.274.5287.546 8849441

[B28] Gasch AP Spellman PT Kao CM Carmel-Harel O Eisen MB Storz G Botstein D Brown PO Genomic expression programs in the response of yeast cells to environmental changes Mole Biology Cell 2000 11 12 4241 4257 10.1091/mbc.11.12.4241 PMC15070 11102521

[B29] Winzeler EA Shoemaker DD Astromoff A Liang H Anderson K Andre B Bangham R Benito R Boeke JD Bussey H Functional characterization of the S. cerevisiae genome by gene deletion and parallel analysis Science (Washington, D C) 1999 285 5429 901 906 10.1126/science.285.5429.901 10436161

[B30] Giaever G Chu AM Ni L Connelly C Riles L Veronneau S Dow S Lucau-Danila A Anderson K Andre B Functional profiling of the Saccharomyces cerevisiae genome Nature 2002 418 6896 387 391 10.1038/nature00935 12140549

[B31] Hughes TR Yeast and drug discovery Functional & Integrative Genomics 2002 2 4–5 199 211 12192593 10.1007/s10142-002-0059-1

[B32] Pryer KM Schneider H Smith AR Cranfill R Wolf PG Hunt JS Sipes SD Horsetails and ferns are a monophyletic group and the closest living relatives to seed plants Nature 2001 409 6820 618 622 10.1038/35054555 11214320

[B33] Veit M Beckert C Hoehne C Bauer K Geiger H Interspecific and intraspecific variation of phenolics in the genus Equisetum subgenus Equisetum Phytochemistry 1995 38 4 881 891 10.1016/0031-9422(94)00658-G

[B34] Gallo FR Multari G Federici E Palazzino G Giambenedetti M Petitto V Poli F Nicoletti M Chemical fingerprinting of Equisetum arvense L. using HPTLC densitometry and HPLC Natural Product Res 2011 25 13 1261 1270 10.1080/14786419.2011.558015 21854173

[B35] Do Monte Fabricio Hoffmann M dos Santos Jair G Jr Russi M Lanziotti Vanusa Maria Nascimento B Leal Luzia Kalyne Almeida M Cunha Geanne Matos De A Antinociceptive and anti-inflammatory properties of the hydroalcoholic extract of stems from Equisetum arvense L. in mice Pharmacol Res 2004 49 3 239 243 10.1016/j.phrs.2003.10.002 14726218

[B36] Safiyeh S Fathallah FB Vahid N Hossine N Habib SS Antidiabetic effect of Equisetum arvense L. (Equisetaceae) in streptozotocin-induced diabetes in male rats Pakistan J Biological Sci: PJBS 2007 10 10 1661 1666 10.3923/pjbs.2007.1661.1666 19086514

[B37] Mimica-Dukic N Simin N Cvejic J Jovin E Orcic D Bozin B Phenolic compounds in field horsetail (Equisetum arvense L.) as natural antioxidants Molecules 2008 13 7 1455 1464 10.3390/molecules13071455 18719517 PMC6245282

[B38] Andrade Cetto A Wiedenfeld H Revilla MC Sergio IA Hypoglycemic effect of Equisetum myriochaetum aerial parts on streptozotocin diabetic rats J Ethnopharmacol 2000 72 1–2 129 133 10967463 10.1016/s0378-8741(00)00218-x

[B39] Perez Gutierrez RM Laguna GY Walkowski A Diuretic activity of Mexican equisetum J Ethnopharmacol 1985 14 2–3 269 272 4094471 10.1016/0378-8741(85)90093-5

[B40] Lemus I Garcia R Erazo S Pena R Parada M Fuenzalida M Diuretic activity of an Equisetum bogotense tea (Platero herb): evaluation in healthy volunteers J Ethnopharmacol 1996 54 1 55 58 10.1016/0378-8741(96)01444-4 8941869

[B41] Schmeda-Hirschmann G Loyola JI Rodriguez J Dutra-Behrens M Hypotensive effect of Laurelia sempervirens (Monimiaceae) on normotensive rats Phytother Res 1994 8 1 49 51 10.1002/ptr.2650080112

[B42] Beckert C Horn C Schnitzler J-P Lehning A Heller W Veit M Styrylpyrone biosynthesis in Equisetum arvense Phytochemistry 1996 44 2 275 283

[B43] Oh H Kim D-H Cho J-H Kim Y-C Hepatoprotective and free radical scavenging activities of phenolic petrosins and flavonoids isolated from Equisetum arvense J Ethnopharmacol 2004 95 2–3 421 424 15507369 10.1016/j.jep.2004.08.015

[B44] D'Agostino M Dini A Pizza C Senatore F Aquino R Sterols from Equisetum arvense Bollettino della Societa italiana di biologia sperimentale 1984 60 12 2241 2245 6529502

[B45] Llorach R Martinez-Sanchez A Tomas-Barberan FA Gil MI Ferreres F Characterization of polyphenols and antioxidant properties of five lettuce varieties and escarole Food Chem 2008 108 3 1028 1038 10.1016/j.foodchem.2007.11.032 26065768

[B46] Amaral JS Ferreres F Andrade PB Valentao P Pinheiro C Santos A Seabra R Phenolic profile of hazelnut (Corylus avellana L.) leaves [of] cultivars grown in Portugal Nat Prod Res 2005 19 2 157 163 10.1080/14786410410001704778 15715260

[B47] Fang N Yu S Prior RL LC/MS/MS Characterization of Phenolic Constituents in Dried Plums J Agric Food Chem 2002 50 12 3579 3585 10.1021/jf0201327 12033832

[B48] Veit M Geiger H Czygan F-C Markham KR Malonylated flavone 5-O-glucosides in the barren sprouts of Equisetum arvense Phytochemistry 1990 29 8 2555 2560 10.1016/0031-9422(90)85187-K

[B49] Plazonic A Bucar F Males Z Mornar A Nigovic B Kujundzic N Identification and quantification of flavonoids and phenolic acids in burr parsley (Caucalis platycarpos L.), using high-performance liquid chromatography with diode array detection and electrospray ionization mass spectrometry Molecules 2009 14 7 2466 2490 10.3390/molecules14072466 19633617 PMC6255260

[B50] Stobiecki M Application of mass spectrometry for identification and structural studies of flavonoid glycosides Phytochemistry 2000 54 3 237 256 10.1016/S0031-9422(00)00091-1 10870178

[B51] Veit M Strack D Czygan FC Wray V Witte L Di-E-caffeoyl-meso-tartaric acid in the barren sprouts of Equisetum arvense Phytochemistry 1991 30 2 527 529 10.1016/0031-9422(91)83720-6

[B52] Rice-Evans CA Miller NJ Paganga G Structure-antioxidant activity relationships of flavonoids and phenolic acids Free Radical Biol Med 1996 20 7 933 956 10.1016/0891-5849(95)02227-9 8743980

[B53] Huang D Ou B Prior RL The chemistry behind antioxidant capacity assays J Agricultural Food Chem 2005 53 6 1841 1856 10.1021/jf030723c 15769103

[B54] Apak R Guclu K Demirata B Ozyurek M Celik SE Bektasoglu B Berker KI Ozyurt D Comparative evaluation of various total antioxidant capacity assays applied to phenolic compounds with the CUPRAC assay Molecules 2007 12 7 1496 1547 10.3390/12071496 17909504 PMC6149428

[B55] Wall ME Rechtsteiner A Rocha LM Singular value decomposition and principal component analysis 2003 Norwell, MA, USA: Kluwer

[B56] Alter O Brown PO Botstein D Singular value decomposition for genome-wide expression data processing and modeling Proc Natl Acad Sci USA 2000 97 18 10101 10106 10.1073/pnas.97.18.10101 10963673 PMC27718

[B57] Brauer MJ Huttenhower C Airoldi EM Rosenstein R Matese JC Gresham D Boer VM Troyanskaya OG Botstein D Coordination of growth rate, cell cycle, stress response, and metabolic activity in yeast Mole Biology Cell 2008 19 1 352 367 10.1091/mbc.E07-08-0779 PMC2174172 17959824

[B58] Smyth G Gentleman R, Irizarry RA, Carey VJ, Dudoit S, Huber W limma: Linear Models for Microarray Data Bioinformatics and Computational Biology Solutions Using R and Bioconductor 2005 New York: Springer Science+Business Media, Inc

[B59] Smyth GK Linear models and empirical bayes methods for assessing differential expression in microarray experiments Stat Applications Genet Mole Biol 2004 3 1 25 10.2202/1544-6115.1027 16646809

[B60] Reimers M Carey VJ Bioconductor: an open source framework for bioinformatics and computational biology Methods Enzymol 2006 411 119 134 16939789 10.1016/S0076-6879(06)11008-3

[B61] Greenberg ML Lopes JM Genetic regulation of phospholipid biosynthesis in Saccharomyces cerevisiae Microbiological Rev 1996 60 1 1 20 10.1128/mr.60.1.1-20.1996 PMC239415 8852893

[B62] White MJ Hirsch JP Henry SA The OPI1 gene of Saccharomyces cerevisiae, a negative regulator of phospholipid biosynthesis, encodes a protein containing polyglutamine tracts and a leucine zipper J Biological Chem 1991 266 2 863 872 1985968

[B63] Ashburner BP Lopes JM Regulation of yeast phospholipid biosynthetic gene expression in response to inositol involves two superimposed mechanisms Proc Natl Acad Sci U S A 1995 92 21 9722 9726 10.1073/pnas.92.21.9722 7568205 PMC40874

[B64] Hirsch JP Henry SA Expression of the Saccharomyces cerevisiae inositol-1-phosphate synthase (INO1) gene is regulated by factors that affect phospholipid synthesis Mole Cell Biol 1986 6 10 3320 3328 10.1128/mcb.6.10.3320-3328.1986 PMC367077 3025587

[B65] Flicek P Amode MR Barrell D Beal K Brent S Carvalho-Silva D Clapham P Coates G Fairley S Fitzgerald S Ensembl 2012 Nucleic Acids Res 2012 40 Database issue D84 D90 22086963 10.1093/nar/gkr991 PMC3245178

[B66] Nicot AS Laporte J Endosomal phosphoinositides and human diseases Traffic 2008 9 8 1240 1249 10.1111/j.1600-0854.2008.00754.x 18429927 PMC2607523

[B67] Gordon PR Mawhinney TP Gilchrest BA Inositol is a required nutrient for keratinocyte growth J Cell Physiology 1988 135 3 416 424 10.1002/jcp.1041350308 2456287

[B68] Lee HJ Lee SA Choi H Dietary administration of inositol and/or inositol-6-phosphate prevents chemically-induced rat hepatocarcinogenesis Asian Pacific J Cancer Prevention : APJCP 2005 6 1 41 47 15780031

[B69] Zeisel SH da Costa KA Choline: an essential nutrient for public health Nutr Rev 2009 67 11 615 623 10.1111/j.1753-4887.2009.00246.x 19906248 PMC2782876

[B70] Canadanovic-Brunet JM Cetkovic GS Djilas SM Tumbas VT Savatovic SS Mandic AI Markov SL Cvetkovic DD Radical scavenging and antimicrobial activity of horsetail (Equisetum arvense L.) extracts Int J Food Sci Technol 2009 44 2 269 278 10.1111/j.1365-2621.2007.01680.x

[B71] Cetojevic-Simin DD Canadanovic-Brunet JM Bogdanovic GM Djilas SM Cetkovic GS Tumbas VT Stojiljkovic BT Antioxidative and antiproliferative activities of different horsetail (Equisetum arvense L.) extracts J Medicinal Food 2010 13 2 452 459 10.1089/jmf.2008.0159 20170379

[B72] Stajner D Popovic BM Canadanovic-Brunet J Anackov G Exploring Equisetum arvense L., Equisetum ramosissimum L. and Equisetum telmateia L. as sources of natural antioxidants Phytotherapy research: PTR 2009 23 4 546 550 10.1002/ptr.2682 19067388

[B73] Gierlinger N Sapei L Paris O Insights into the chemical composition of Equisetum hyemale by high resolution Raman imaging Planta 2008 227 5 969 980 10.1007/s00425-007-0671-3 18057960 PMC2756348

[B74] Dos Santos JG Jr Blanco MM Do Monte FH Russi M Lanziotti VM Leal LK Cunha GM Sedative and anticonvulsant effects of hydroalcoholic extract of Equisetum arvense Fitoterapia 2005 76 6 508 513 10.1016/j.fitote.2005.04.017 15972249

[B75] Guilherme dos Santos J Jr Hoffmann Martins do Monte F Marcela Blanco M Maria do Nascimento Bispo Lanziotti V Damasseno Maia F Kalyne de Almeida Leal L Cognitive enhancement in aged rats after chronic administration of Equisetum arvense L. with demonstrated antioxidant properties in vitro Pharmacol Biochem Behav 2005 81 3 593 600 10.1016/j.pbb.2005.04.012 15972233

[B76] Graefe EU Veit M Urinary metabolites of flavonoids and hydroxycinnamic acids in humans after application of a crude extract from Equisetum arvense Phytomedicine: International J Phytotherapy Phytopharmacology 1999 6 4 239 246 10.1016/S0944-7113(99)80015-4 10589442

[B77] Wang CY Staniforth V Chiao MT Hou CC Wu HM Yeh KC Chen CH Hwang PI Wen TN Shyur LF Genomics and proteomics of immune modulatory effects of a butanol fraction of echinacea purpurea in human dendritic cells BMC genomics 2008 9 479 10.1186/1471-2164-9-479 18847511 PMC2571112

[B78] Wang E Bussom S Chen J Quinn C Bedognetti D Lam W Guan F Jiang Z Mark Y Zhao Y Interaction of a traditional Chinese Medicine (PHY906) and CPT-11 on the inflammatory process in the tumor microenvironment BMC Med Genomics 2011 4 38 10.1186/1755-8794-4-38 21569348 PMC3117677

[B79] Qin S Chen J Tanigawa S Hou DX Gene expression profiling and pathway network analysis of hepatic metabolic enzymes targeted by baicalein J Ethnopharmacology 2012 140 1 131 140 10.1016/j.jep.2011.12.046 22265932

[B80] Rong J Tilton R Shen J Ng KM Liu C Tam PK Lau AS Cheng YC Genome-wide biological response fingerprinting (BioReF) of the Chinese botanical formulation ISF-1 enables the selection of multiple marker genes as a potential metric for quality control J Ethnopharmacology 2007 113 1 35 44 10.1016/j.jep.2007.01.021 17307317

[B81] Valente AX Roberts SB Buck GA Gao Y Functional organization of the yeast proteome by a yeast interactome map Proc Natl Acad Sci USA 2009 106 5 1490 1495 10.1073/pnas.0808624106 19164585 PMC2635830

[B82] Griffin TJ Gygi SP Ideker T Rist B Eng J Hood L Aebersold R Complementary profiling of gene expression at the transcriptome and proteome levels in Saccharomyces cerevisiae Mole & Cellular Proteomics: MCP 2002 1 4 323 333 10.1074/mcp.M200001-MCP200 12096114

[B83] Rossouw D Naes T Bauer FF Linking gene regulation and the exo-metabolome: a comparative transcriptomics approach to identify genes that impact on the production of volatile aroma compounds in yeast BMC Genomics 2008 9 530 10.1186/1471-2164-9-530 18990252 PMC2585593

[B84] Mazzocchi F Complexity and the reductionism-holism debate in systems biology Wiley interdisciplinary reviews Systems biology and medicine 2012 4 5 413 427 10.1002/wsbm.1181 22761024

[B85] Van Regenmortel MH Reductionism and complexity in molecular biology Scientists now have the tools to unravel biological and overcome the limitations of reductionism. EMBO reports 2004 5 11 1016 1020 10.1038/sj.embor.7400284 PMC1299179 15520799

[B86] Hollingsworth PM Forrest LL Spouge JL Hajibabaei M Ratnasingham S van der Bank M Chase MW Cowan RS Erickson DL Fazekas AJ A DNA barcode for land plants Proc Natl Acad Sci U S A 2009 106 31 12794 12797 S12794/12791-S12794/12736 19666622 10.1073/pnas.0905845106 PMC2722355

[B87] DNA barcoding phase 2 Updatehttp://www.barcoding.si.edu/plant_working_group.html

[B88] Wagner H Bladt S Plant drug analysis : a thin layer chromatography atlas, 2nd edn. Dordrecht 2009 New York: Springer

[B89] Bandoniene D Murkovic M On-line HPLC-DPPH screening method for evaluation of radical scavenging phenols extracted from apples (Malus domestica L.) J Agric Food Chem 2002 50 9 2482 2487 10.1021/jf011475s 11958609

[B90] Blois MS Antioxidant Determinations by the Use of a Stable Free Radical Nature 1958 181 1199 1200 10.1038/1811199a0

[B91] Molyneux P The use of the stable free radical diphenylpicrylhydrazyl (DPPH) for estimating antioxidant activity.*Songklanakarin J* Part Sci Technol 2004 26 211 219

[B92] LABTECH B ORAC Assay on the FLUOstar OPTIMA to Determine Antioxidant Capacity http://www.bmglabtech.com/application-notes/fluorescence-intensity/orac-148.cfm

[B93] Baker Brachmann C Davies A Cost GJ Caputo E Li J Hieter P Boeke JD Designer deletion strains derived from Saccharomyces cerevisiae S288C: A useful set of strains and plasmids for PCR-mediated gene disruption and other applications Yeast 1998 14 2 115 132 10.1002/(SICI)1097-0061(19980130)14:2<115::AID-YEA204>3.0.CO;2-2 9483801

[B94] Alic N Felder T Temple MD Gloeckner C Higgins VJ Briza P Dawes IW Genome-wide transcriptional responses to a lipid hydroperoxide: adaptation occurs without induction of oxidant defenses Free Radical Biology and Medicine 2004 37 1 23 35 10.1016/j.freeradbiomed.2004.04.014 15183192

[B95] Bell PJL Higgins VJ Dawes IW Bissinger PH Tandemly Repeated 147 bp Elements Cause Structural and Functional Variation in Divergent MAL Promoters of Saccharomyces cerevisiae Yeast 1997 13 12 1135 1144 10.1002/(SICI)1097-0061(19970930)13:12<1135::AID-YEA162>3.0.CO;2-1 9301020

[B96] R Core Development Team A Language Environment for Statistical Computing A Language Environment for Statistical Computing 2010 Vienna: Foundation for Statistical Computing

[B97] Gong L Constantine W Chen YA msProcess: Protein Mass Spectra Processing R Package, version 106 2011

[B98] Software GP Prism 5 for Mac OS X. In 2009 GraphPad Software: San Diego

[B99] Gentleman RC Carey VJ Bates DM Bolstad B Dettling M Dudoit S Ellis B Gautier L Ge Y Gentry J Bioconductor: open software development for computational biology and bioinformatics Genome Biol 2004 5 10 R80 10.1186/gb-2004-5-10-r80 15461798 PMC545600

[B100] Gautier L Cope L Bolstad BM Irizarry RA affy-analysis of Affymetrix GeneChip data at the probe level Bioinformatics 2004 20 3 307 315 10.1093/bioinformatics/btg405 14960456

[B101] Bolstad BM Collin F Brettschneider J Simpson K Cope L Irizarry RA Speed TP Gentleman R, Irizarry RA, Carey VJ, Dudoit S, Huber W Quality Assessment of Affymetrix GeneChip Data Bioinformatics and Computational Biology Solutions Using R and Bioconductor 2005 New York: Springer Science+Business Media, Inc

[B102] Gautier L Møller M Friis-Hansen L Knudsen S Alternative mapping of probes to genes for Affymetrix chips BMC Bioinformatics 2004 5 111 10.1186/1471-2105-5-111 15310390 PMC514699

[B103] Smith C Smith C Smith CA annaffy: Annotation tools for Affymetrix biological metadata R package version 1.32.0 2010

[B104] The Bioconductor Project: yeast2cdf: yeast2cdf yeast2cdf: yeast2cdf R package version 2.12.0

[B105] Carlson M Falcon S Pages H Li N yeast2.db: Affymetrix Yeast Genome 2.0 Array annotation data (chip yeast2) R package version 2.9.0

[B106] Irizarry R Hobbs B Collin F Beazer-Barclay Y Antonellis K Scherf U Speed T Exploration, normalization, and summaries of high density oligonucleotide array probe level data Biostatistics 2003 4 2 249 264 10.1093/biostatistics/4.2.249 12925520

[B107] Hennell JR Quality control methods for herbal medicine: a multifaceted approach 2012 University of Western Sydney

[B108] Robinson MD Grigull J Mohammad N Hughes TR FunSpec: a web-based cluster interpreter for yeast BMC bioinformatics 2002 3 35 10.1186/1471-2105-3-35 12431279 PMC139976

[B109] Kanehisa M Goto S Sato Y Furumichi M Tanabe M KEGG for integration and interpretation of large-scale molecular data sets Nucleic Acids Res 2012 40 Database issue D109 114 22080510 10.1093/nar/gkr988 PMC3245020

[B110] Cherry JM Hong EL Amundsen C Balakrishnan R Binkley G Chan ET Christie KR Costanzo MC Dwight SS Engel SR Saccharomyces Genome Database: the genomics resource of budding yeast Nucleic Acids Res 2012 40 Database issue D700 705 22110037 10.1093/nar/gkr1029 PMC3245034

