# Supplementary material for: The Saccharomyces cerevisiae transcriptome as a mirror of phytochemical variation in complex extracts of Equisetum arvense from America, China, Europe and India
Source: BMC Genomics. 2013 Jul 4;14:445. doi: 10.1186/1471-2164-14-445 (PMC3720287; doi:10.1186/1471-2164-14-445)
Supplement: Additional file 1: Figure S1 — Tentative structural elucidation of dicaffeoyltartaric (chicoric) acid and a genkwanin acetylglucoside using LC-ESI(-)-MS and LC-PDA. (A, B, C) the ESI(-)-MS, UV spectrum and proposed fragmentation pattern respectively of the dicaffeoyltartaric acid peak. (D, E, F) the ESI(-)-MS, UV spectrum and proposed fragmentation pattern respectively of the Genkwanin acetylglucoside peak, possibly 4 or 5 -O-(6-acetyl glucoside). [file 1471-2164-14-445-S1.pdf]

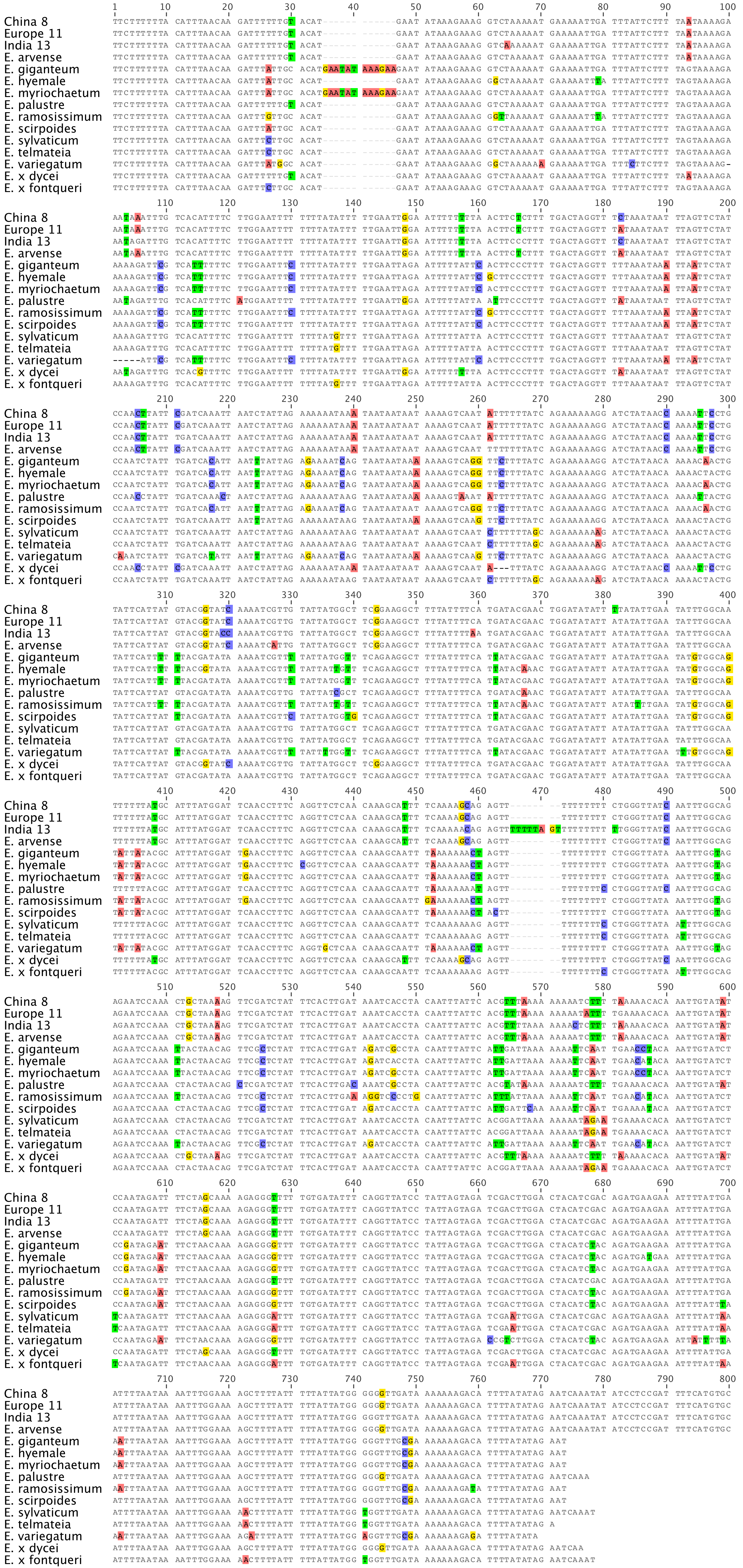

China 8
Europe 11
India 13
E. arvense
E. giganteum
E. hyemale
E. myriochaetum
E. palustre
E. ramosissimum
E. scirpoides
E. sylvaticum
E. telmateia
E. variegatum
E. x dycei
E. x fontqueri
1 10 20 30 40 50 60 70 80 90 100
110 120 130 140 150 160 170 180 190 200
210 220 230 240 250 260 270 280 290 300
310 320 330 340 350 360 370 380 390 400
410 420 430 440 450 460 470 480 490 500
510 520 530 540 550 560 570 580 590 600
610 620 630 640 650 660 670 680 690 700
710 720 730 740 750 760 770 780 790 800
