# Supplementary figures and images for: The Saccharomyces cerevisiae transcriptome as a mirror of phytochemical variation in complex extracts of Equisetum arvense from America, China, Europe and India
Source: BMC Genomics. 2013 Jul 4;14:445. doi: 10.1186/1471-2164-14-445 (PMC3720287; doi:10.1186/1471-2164-14-445)

**A**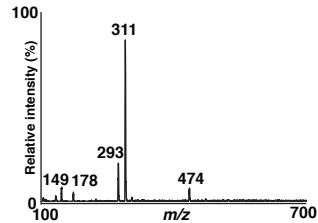**B**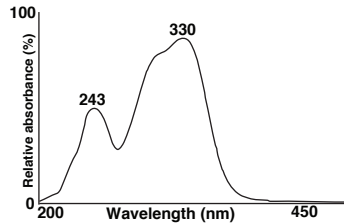**D**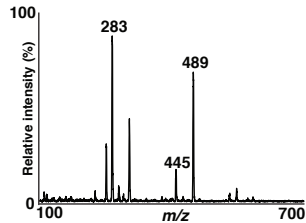**E**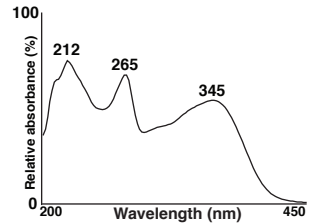**C**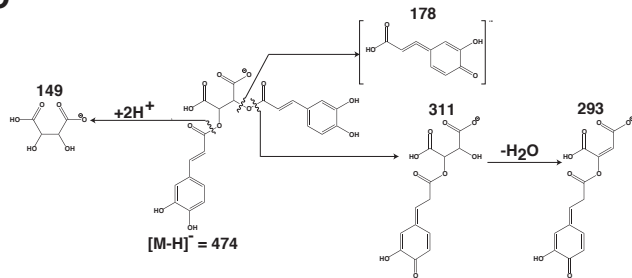**F**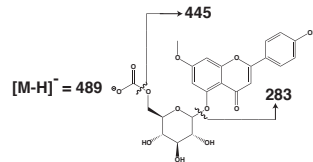

Supplement: Additional file 2: Figure S2 — DNA bar codes of the original plant material used to produce the China 8, Europe 11 and India 13 extracts compared to other Equisetum species entries in the GenBank database. Differences between the sequences are marked with a colored box. [file 1471-2164-14-445-S2.pdf]
